# Chinese primary school physical education teachers’ ongoing training and professional competence: an evidence mapping review and a hypothesis-generating conceptual framework

**DOI:** 10.3389/fpsyg.2026.1901909

**Published:** 2026-08-10

**Authors:** Heng Li, Bing Shi

**Affiliations:** 1 School of Teacher Development, Shaanxi Normal University, Xi’an, China; 2 Kangbashiqu Experimental Primary School, Ordos, Inner Mongolia, China; 3 Physical Education School, Shaanxi Normal University, Xi’an, China

**Keywords:** ongoing training, physical education, primary teachers, professional competence, professional development

## Abstract

Ongoing training has gained increasing research attention as a means to improve the professional competencies of Chinese primary school physical education (PE) teachers. However, prior reviews have largely focused on general teachers, secondary teachers, or other subjects, leaving primary school PE teachers under-researched. Following the Preferred Reporting Items for Systematic Reviews and Meta-Analyses (PRISMA) 2020 guidelines, we systematically searched Web of Science and Scopus, identifying 29 peer-reviewed studies. Methodological quality was assessed using the Mixed Methods Appraisal Tool (MMAT), and findings were synthesized thematically. Critically, no included study directly targeted Chinese primary school PE teachers, revealing a fundamental evidence gap. Thematic synthesis of indirect evidence from adjacent populations, including general primary teachers, secondary teachers, teachers of other subjects, and non-Chinese contexts, identified four competency dimensions that may be influenced by ongoing training, namely instructional design, professional knowledge, teaching practice, and self-efficacy. Given the complete absence of direct evidence, all conclusions drawn for the target population are explicitly hypothesis-generating rather than confirmatory. Based on these indirect findings, we tentatively propose a testable chain mediation model in which ongoing training may sequentially influence professional knowledge, teaching practice, self-efficacy, and ultimately professional competence, offering a potential framework to guide future empirical investigations. This mini-review contributes an evidence map for this under-researched population and provides a heuristic conceptual framework to inform future research designs.

## 1 Introduction

As the Chinese reformer Tao Xingzhi stated, “Living, doing, learning, and teaching are lifelong endeavors; only upon entering the coffin can one be considered graduated” (Xu and Xu, 2010, p. 193). This philosophy underscores that educators’ professional growth is a continuous process requiring ongoing renewal of pedagogical knowledge and skills. In China, improving teachers’ professional competencies through sustained professional development has received increasing research attention. Various interventions have been proposed to address teachers’ difficulties in implementing innovative teaching models and adapting to curriculum reforms (Butnariu and Roman, 2025; Desimone, 2009; Greitans and Namsone, 2024; Ha et al., 2015).

However, although several reviews have examined teacher professional development in China, none has focused specifically on ongoing training for primary school PE teachers. Musengamana and Hou (2025) concentrated on secondary teachers. Ma (2025) excluded PE specialists. Other reviews targeted non-PE or secondary contexts (Wu and Shen, 2024; Zhang and Wong, 2021). These prior reviews have thus left primary school PE teachers under-researched, despite their unique challenges. These challenges include outdoor class management, injury response, and large-class motor skill instruction, which distinguish them from teachers of other subjects or levels.

Recently, researchers have called for more empirical and review studies on teacher professional development in under-researched subject areas, particularly physical education in primary schools (Yan and Dai, 2025). In response, this mini-review aims to make two distinctive contributions. First, it operationalizes the construct of ongoing training specifically for the PE context, synthesizing how it has been conceptualized across existing studies. Second, drawing on thematic synthesis of indirect evidence, it proposes a hypothesized chain mediation model in which ongoing training may influence professional competence sequentially through professional knowledge, teaching practice, and self-efficacy, offering a testable framework for future research.

Accordingly, this mini-review is guided by two research questions: (1) How is ongoing training conceptualized in the existing literature? and (2) What does the available indirect evidence suggest about potential pathways through which ongoing training might relate to professional competence, and what testable hypotheses can be generated for future empirical research on Chinese primary PE teachers?

Importantly, direct empirical studies on Chinese primary PE teachers are expected to be scarce. Therefore, this mini-review adopts a broad-scope evidence mapping strategy that includes studies from adjacent populations (e.g., other subjects, secondary levels, and non-Chinese contexts) to identify evidence gaps and generate hypotheses for the target population. All inferences drawn for Chinese primary PE teachers are therefore presented as hypothesis-generating rather than confirmatory. This evidence gap, however, is not merely a limitation but a central finding of this review.

## 2 Definition of terms

Before data collection, defining key terms helps reduce false-positive results (Cardiff et al., 2023). Following Musengamana and Hou (2025, p. 3), we use “on-the-job training” and “ongoing training” interchangeably to refer to all forms of professional development activities that in-service teachers participate in. These include induction programs, seminars, workshops, mentoring, peer observation, collaboration, online learning, school-based research, and reflective practice.

The multidimensional framework of Kunter et al. (2013) provides a comprehensive theoretical lens. However, in the empirical literature on ongoing training, professional beliefs and self-regulatory characteristics (e.g., reflection) are inconsistently operationalized or rarely measured as dependent variables, precluding meaningful synthesis. Therefore, we focused on the four dimensions that are (a) most frequently and consistently measured across the included studies, (b) most directly amenable to training interventions, and (c) aligned with primary PE teaching demands: instructional design, professional knowledge, teaching practice, and self-efficacy. We acknowledge that this selective focus narrows the theoretical scope; this limitation is revisited in Section 5.

## 3 Methods

### 3.1 Types/sources of data

#### 3.1.1 Rationale for evidence mapping

This review adopts an evidence mapping approach, which differs from traditional systematic reviews in two fundamental ways. First, whereas systematic reviews typically aim to aggregate effect sizes to answer a specific causal question (e.g., ‘How large is the effect of training on competence?’), evidence mapping is designed to systematically identify, categorize, and visualize the volume, distribution, and gaps in the existing evidence base for a broad, under-researched topic (Arksey and O’Malley, 2005). Second, unlike scoping reviews, which often provide a narrative overview without formal synthesis, evidence mapping in this study is followed by thematic synthesis to generate theoretical propositions for future testing. Thus, this methodology is appropriate because the primary aim is to map evidence gaps and generate hypotheses, not to produce confirmatory conclusions.

#### 3.1.2 Search strategy

This mini-review adheres to the PRISMA 2020 statement (Page et al., 2021). The flow diagram (Supplementary Figure S1) and checklist (Supplementary Table S2) are provided in Supplementary material.

We searched Scopus and Web of Science on May 5, 2026 for peer-reviewed articles related to ongoing training, physical education teachers, China, primary school and professional competence. All search terms were derived from validated keyword variants used in prior peer-reviewed studies on teacher professional development, as cited below.

For ongoing training, we included on-going training, on-going education, and continuing education, following O’Donovan et al. (2018); Learn*, train*, and educat*, drawing on Ramachandran et al. (2024). For professional competence, we included professional development and instructional leadership, following Rahman and Yuliani (2025); teacher professional development and teacher competence, based on Marnoko (2025); teacher competency development, following Nasrullah and Arhas (2025); competence, competency, core competence, and core competency, as defined by Yaqoob Mohammed Al Jabri et al. (2021); and teaching competence, literacy, and skill, following Wang and Sang (2024).

To balance sensitivity and specificity, we used a Boolean combination requiring each record to contain at least one term from each conceptual group (training-related AND competence-related AND context-related) in the title/abstract fields, reducing noise from overly broad terms while maintaining sensitivity. The full search syntax, including all Boolean operators, is provided as Supplementary Table S3.

We applied the title and abstract fields in the search. Our initial search identified 1,074 articles in Web of Science and 1,442 in Scopus, which were imported into Zotero reference management software. Of these 2,516 articles, 724 were identified as duplicates, and 6 were removed for other reasons, leaving 1,786 records for screening.

#### 3.1.3 Inclusion and exclusion

To manage the anticipated absence of direct evidence, we established a tiered eligibility framework.

Primary (direct) eligibility: Studies were included if they (a) were empirical peer-reviewed articles, (b) examined ongoing training or professional development, (c) measured at least one dimension of professional competence, and (d) specifically targeted in-service primary school physical education teachers in China.

Secondary (indirect) eligibility: Recognizing that primary-tier studies were expected to be scarce, we also included studies that met all criteria above but targeted adjacent populations, including: (a) Chinese primary school teachers of other subjects (e.g., mathematics, Chinese, English), (b) Chinese secondary school PE teachers, (c) Chinese teachers of other disciplines, and (d) non-Chinese PE teachers or general theoretical models of teacher competence.

Analytical distinction: Studies from the primary tier (direct evidence) and secondary tier (indirect evidence) were analyzed separately. Findings from indirect evidence are explicitly labeled as such throughout the Results and Discussion, and all inferences for Chinese primary PE teachers are presented as hypothesis-generating.

Study selection was conducted independently by two reviewers, with disagreements resolved through discussion. We screened 1,786 records after deduplication. We excluded 43 non-English articles, 173 conference papers, and 25 books, leaving 1,545 reports for full-text retrieval. Full texts were retrieved for all reports. During eligibility assessment, we excluded 1,519 reports unrelated to ongoing training and professional competence, along with 2 correction notices, 2 literature abstracts, and 1 opinion article. We then identified 8 additional studies through reference list scanning and Google Scholar. This yielded 29 studies in the final review sample. Supplementary Table S1 provides detailed exclusion reasons with examples (e.g., non-PE subject, non-teacher sample, no training variable).

#### 3.1.4 Quality appraisal

To enhance the credibility of our synthesis, we assessed the methodological quality of all 29 included studies using the MMAT (Hong et al., 2018). We selected MMAT because it is suitable for appraising qualitative, quantitative, and mixed-methods studies, all of which are present in our sample. Two reviewers independently rated each study as “high,” “moderate,” or “low” quality based on the relevant MMAT criteria. Discrepancies were resolved by discussion. The quality ratings are presented alongside study characteristics in Supplementary Table S4. Although all studies were retained in the synthesis, we explicitly considered quality ratings when interpreting the strength of the evidence.

### 3.2 Extraction and analysis

We extracted data from each included study following the procedure proposed by Yang and Whatman (2025). Extracted parameters included author(s)/year of publication, country of study, study design, study participants, data collection approach, analysis methods, and results/findings, which are summarized in Supplementary Table S4.

## 4 Findings and discussion

### 4.1 Evidence mapping and study characteristics

A total of 29 studies were included. Based on their relevance to our target population (Chinese primary school PE teachers), we classified them into three tiers: Tier 1 (Direct evidence): Studies exactly targeting Chinese primary school PE teachers. Count: 0. Tier 2 (Adjacent evidence): Studies on Chinese primary school teachers of other subjects (e.g., English, Chinese, Math), or Chinese secondary school PE teachers. Count: 18 (e.g., Ha et al., 2004; Musengamana and Hou, 2025; Wang et al., 2023; Wang and Fan, 2025; Wu and Shen, 2024). Tier 3 (Indirect/general evidence): Studies on non-Chinese contexts, higher education (college) PE teachers, or general theoretical models. Count: 11 (e.g., Chen, 2025 on AI systems; Kunter et al., 2013 for Germany; Li, 2023 for the Philippines; Opfer and Pedder, 2011 for the UK; Rozi et al., 2025 for Indonesia).

**Figure 1:**
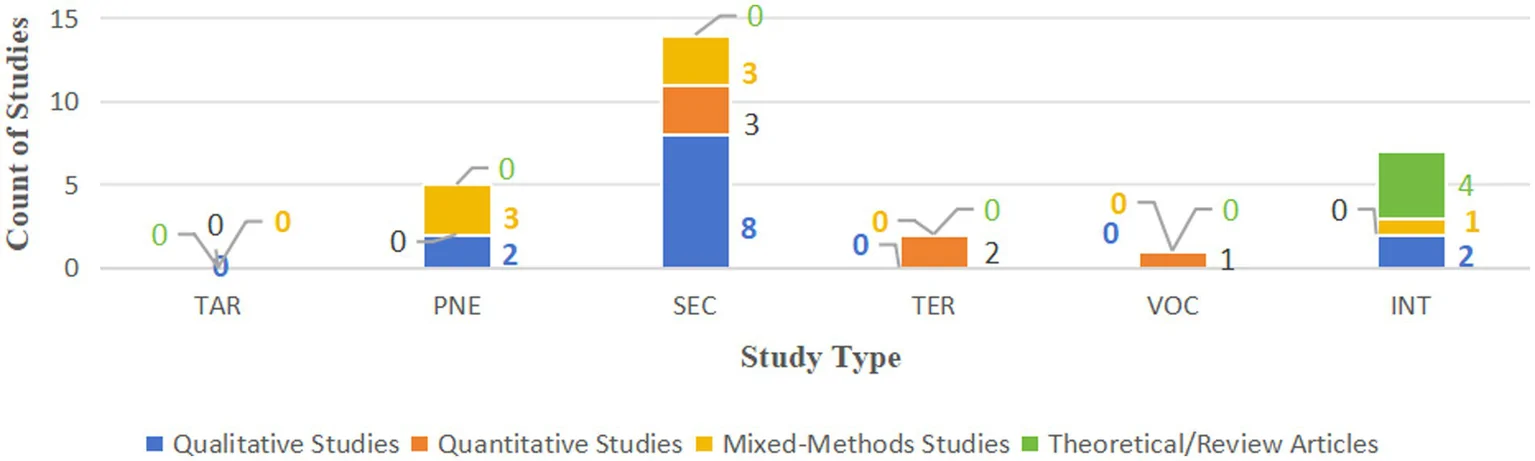
Distribution of the 29 included studies by education level and study design (*N* = 29). TAR, Chinese primary school PE teachers (target population); PNE, Chinese primary school teachers of other subjects; SEC, Chinese secondary school teachers; TER, Chinese tertiary/college PE teachers; VOC, Chinese vocational or cross-level teacher samples; INT, Studies conducted in non-Chinese contexts.

This mapping reveals a critical evidence gap: in our sample, no English-language peer-reviewed study has directly examined ongoing training for Chinese primary school PE teachers. Consequently, all findings presented below are hypothesis-generating inferences drawn from adjacent and indirect evidence, rather than confirmatory conclusions for the target population. As visualized in Figure 1, the evidence gap is most pronounced at the intersection of primary school, PE subject, and Chinese context, where zero studies were identified.

### 4.2 Thematic synthesis: how ongoing training is conceptualized

Across the 29 studies, ongoing training was consistently portrayed not as a one-off event, but as an embedded, sustained professional learning process (Gu and Wang, 2006; Wong and Tsui, 2007; Yan and He, 2015). This conceptualization emerged from both theoretical models of teacher learning and empirical investigations of in-service training programs.

Four core components were repeatedly identified:

1. Formal structured programs (workshops, short courses, TKT-based training) (e.g., Ding et al., 2023; Wang et al., 2023).
2. School-based embedded activities (collective lesson preparation, open classes, peer observation, mentoring) (e.g., Han and Feng, 2013; Wu and Shen, 2024; Zhang and Wong, 2021).
3. Collaborative professional communities (PLCs, communities of practice) (e.g., Sargent and Hannum, 2009; Wang and Fan, 2025).
4. Technology-enhanced or data-driven models (AI recommendation systems, IoT-based training) (Luo et al., 2026; Rozi et al., 2025). Complementarily, reflective practice serves as a critical bridge between knowledge and classroom enactment (Bjørke and Quennerstedt, 2026).

Notably, these components are interdependent. For example, school-based activities (component 2) often serve as the implementation ground for formal training (component 1), while PLCs (component 3) provide the social infrastructure for sustained reflection. This synthesis moves beyond a simple definition to a multi-component conceptualization, which we later integrate into a theoretical model (see Section 4.4).

### 4.3 Thematic synthesis: effects on professional competencies

We synthesized findings by four competency dimensions: instructional design, professional knowledge, teaching practice, and self-efficacy, with evidence strength, consistency, and tier reported for each.

#### 4.3.1 Instructional design

Evidence from the included studies suggests that ongoing training, particularly school-based lesson study and open-class activities, enhances teachers’ ability to design age-appropriate and goal-oriented lessons. Wu and Shen (2024) conducted a qualitative study of 19 teachers and found that the iterative cycle of rehearsing, peer feedback, and revision during open class preparation improved instructional design quality. Similarly, Han and Feng (2013) reported that school-based instructional research helped teachers align lesson plans with China’s New Curriculum standards. However, no study directly measured instructional design among Chinese primary PE teachers; thus, this finding is a transferable inference drawn from adjacent populations.

#### 4.3.2 Professional knowledge

Both formal training and school-based learning were reported to expand teachers’ content knowledge and pedagogical content knowledge (PCK). Zhang and Wong (2021) showed that secondary teachers accumulated “local knowledge” (context-specific teaching strategies) through peer observation and mentoring. Musengamana and Hou (2025), in a large-scale survey of 3,976 secondary teachers, found significant positive effects of on-the-job training on human capital (operationalized as professional knowledge and skills). In the PE-specific context, Ha et al. (2004) reported that primary teachers (generalists teaching PE) gained knowledge about curriculum implementation after in-service training, though the study lacked a control group. Notably, Rozi et al. (2025) found that a digital training model enhanced Indonesian PE teachers’ professional knowledge, suggesting that technology-mediated training may be a promising avenue for Chinese primary PE contexts.

#### 4.3.3 Teaching practice

Evidence from Tier 2 and Tier 3 suggests that ongoing training may facilitate the translation of knowledge into classroom practice. Wang and Fan (2025), in a longitudinal mixed-methods study of 135 primary and secondary teachers (including PE teachers), found that communities of practice significantly enhanced professional development through improved classroom strategies. Opfer and Pedder (2011) conceptual review argued that effective professional development must be sustained and intensive to change instructional practices, a view consistent with the ethnographic study by Yan and Dai (2025), who emphasized that fragmented training fails to alter daily teaching routines.

Furthermore, reflective practice serves as a critical bridge between knowledge and classroom enactment. Bjørke and Quennerstedt (2026) argued that structured reflection on embodied and situated PE experiences helps students move beyond the theory-practice divide. This finding underscores the value of incorporating reflective components in teacher professional development to support the translation of knowledge into practice.

#### 4.3.4 Self-efficacy

Self-efficacy emerged as the most frequently measured and consistently enhanced outcome. Musengamana and Hou (2025) reported a strong direct effect of on-the-job training on teacher self-efficacy (*p* < 0.001). Wang et al. (2023) provided rich qualitative evidence from primary English teachers, showing that mastery experiences during a 4-month CPD program shifted teachers’ beliefs from resistance to confidence. Ha et al. (2004) similarly found that primary teachers felt “more secure and confident” after collaborative in-service training. Within PE, Yan and Dai (2025) noted that rural PE teachers’ self-efficacy was mediated by school culture and collegial support, suggesting that training alone may be insufficient without institutional backing.

Beyond the four dimensions synthesized above, recent empirical evidence highlights that physical education teachers themselves perceive continuous professional development and evaluation as integral components of their professional competence and lifelong learning trajectories (Stafylidis et al., 2024). This finding, derived from vocational education and training contexts, lends cross-situational support to our framework by underscoring that competence is not merely a static knowledge set but is dynamically shaped by engagement with evaluation and ongoing learning.

### 4.4 A synthesized conceptual framework: the chain mediation model

**Figure 2:**
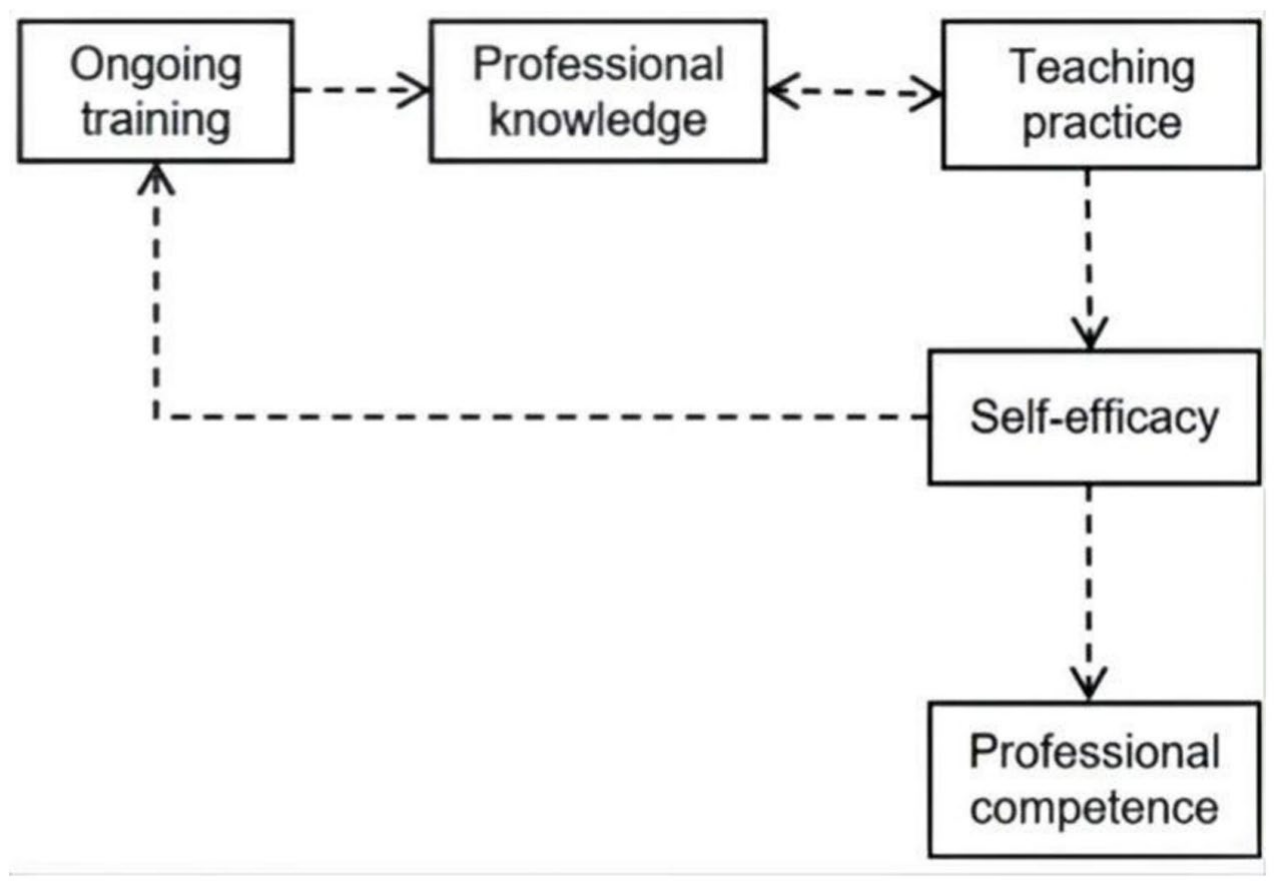
Conceptual framework: Hypothesized chain mediation model of ongoing training and professional competence. Dashed arrows denote hypothetical pathways, not confirmed causality. The bidirectional arrow between professional knowledge and teaching practice reflects their reciprocal relationship (Bjørke and Quennerstedt, 2026). The reverse arrow from self-efficacy to ongoing training acknowledges that efficacious teachers may seek more development. The model includes four competency dimensions most consistently measured; professional beliefs and self-regulation were excluded due to inconsistent operationalization (see Section 2). This framework is heuristic, not deterministic. Competing models (e.g., efficacy as a precursor) warrant future testing.

Based on the thematic synthesis above, we tentatively propose a testable conceptual framework that integrates the fragmented evidence (see Figure 2 for a visual model). The framework specifies a sequential pathway: ongoing training influences professional knowledge, which in turn shapes teaching practice, and this enhanced practice subsequently strengthens self-efficacy, ultimately leading to improved professional competence.

The rationale is as follows. We present this sequential pathway as a heuristic device for hypothesis generation, not as a deterministic causal chain. Alternative directions are theoretically plausible: for instance, teachers with higher self-efficacy may seek out more training opportunities, and teaching practice may reciprocally refine professional knowledge through reflection (Bjørke and Quennerstedt, 2026). The proposed ordering reflects the typical temporal sequence from formal training inputs (knowledge acquisition) through behavioral enactment (practice) to motivational internalization (efficacy), but future empirical studies should test competing path models (e.g., efficacy as a precursor).

Ongoing training, whether formal, school-based, collaborative, or technology-enhanced, serves as the initial stimulus that generates potential cognitive gains in professional knowledge, which is then translated into teaching practice through school-based application opportunities. This practical application subsequently strengthens self-efficacy through mastery experiences in real classrooms, ultimately contributing to the integrative endpoint of professional competence: the knowledge, skills, and beliefs required for effective PE teaching. This conceptualization aligns with the multidimensional framework of Kunter et al. (2013), which defines professional competence as encompassing knowledge, beliefs, and motivational and self-regulatory characteristics. These pathways are supported by empirical evidence: the knowledge acquisition pathway (Musengamana and Hou, 2025; Zhang and Wong, 2021), the behavioral enactment pathway (Wang and Fan, 2025; Wu and Shen, 2024), and the efficacy enhancement pathway (Ha et al., 2004; Wang et al., 2023).

This framework makes three potential theoretical contributions: (a) it specifies a causal sequence rather than treating competencies as parallel correlates; (b) it identifies self-efficacy as a critical mediating mechanism rather than a standalone outcome; and (c) it provides a heuristic tool for future empirical research on Chinese primary PE teachers.

### 4.5 Evidence gaps and methodological limitations of the included studies

Our synthesis of the 29 studies revealed persistent limitations that constrain the validity of our inferences:

Absence of direct evidence: No study directly targeted Chinese primary school PE teachers (see Section 4.1). This was the most critical gap identified.

Predominance of self-reported data: More than 80% of studies relied on surveys, interviews, or reflective journals, introducing social desirability and recall biases. Only Chen (2025) and Luo et al. (2026) used multimodal or IoT-based objective measures, but these were conducted with college PE teachers.

Weak causal designs: Few studies employed randomized controlled trials or pre-test/post-test with control groups. Ha et al. (2004) used a single-cohort design; Musengamana and Hou (2025) used cross-sectional surveys; none established causality.

Additionally, while Chen (2025) and Luo et al. (2026) offer illustrative examples of technology-enhanced training models, both studies were conducted with college rather than primary PE teachers and appeared in journals with broad, interdisciplinary scopes. Given their limited contextual relevance and potential source-specific biases, we treat these as exploratory technical precedents rather than foundational evidence for our framework.

Geographic and language bias: The majority of studies were conducted in China, limiting cross-cultural generalizability. Additionally, our reliance on English-language databases may have excluded relevant Chinese-language studies published in local journals, introducing language bias.

Publication bias: The included studies predominantly reported positive effects. Null or negative findings on ongoing training are likely underrepresented in the peer-reviewed literature.

Quality heterogeneity: While we assessed methodological quality using MMAT, the overall evidence base was of moderate to low quality, with few studies meeting all quality criteria.

We acknowledge that these limitations mean our findings should be interpreted as hypothesis-generating, not confirmatory. The proposed conceptual framework (see Section 4.4) is a tentative model that requires empirical testing with rigorous designs targeting the specific population of Chinese primary school PE teachers.

## 5 Conclusion and limitation

This mini-review maps the existing evidence base on ongoing training and professional competence in the context of Chinese primary school physical education, and reveals a critical finding: no direct empirical study has examined this relationship in the target population. All 29 included studies were derived from adjacent populations, including general primary teachers, secondary school teachers, teachers of other subjects, and non-Chinese contexts. Drawing on indirect evidence from these adjacent populations, we propose a tentative, hypothesis-generating conceptual framework that specifies a sequential pathway through which ongoing training may relate to instructional design, professional knowledge, teaching practice, and self-efficacy. This framework is not intended as a confirmatory model, but as a heuristic tool to guide future empirical research specifically targeting Chinese primary school PE teachers.

This framework synthesizes fragmented evidence into a testable model and specifies how training may influence competence through three mechanisms: cognitive (professional knowledge), behavioral (teaching practice), and motivational (self-efficacy).

We acknowledge several limitations. Most included studies used cross-sectional designs and self-reported data, limiting causal inference. The predominance of positive findings suggests likely publication bias, and the geographic concentration in China limits the cross-cultural generalizability of our findings. Additionally, our reliance on Scopus and Web of Science may have excluded relevant Chinese-language studies. Our reliance on a few studies published in emerging or broad-scope journals (e.g., Chen, 2025; Luo et al., 2026) introduces source heterogeneity; we have therefore restricted their weight in theory building. Furthermore, given the indirect nature of our evidence base, our inferences for Chinese primary PE teachers are vulnerable to ecological fallacy—that is, assuming relationships observed in other populations automatically apply to the target group. Given these limitations, our findings should be interpreted as hypothesis-generating rather than confirmatory.

Despite these limitations, we have enhanced transparency by using literature-derived search terms and providing detailed characteristics for all 29 included articles. We also offer tentative practice recommendations: establishing PE-focused Professional Learning Communities, implementing structured peer-coaching, embedding school-based action research, and leveraging AI-based tools for personalized feedback. These recommendations may be particularly relevant for resource-constrained rural schools.

Future research could proceed through three stages: descriptive surveys to map current training participation patterns, qualitative inquiries to understand PE-specific learning pathways, and quasi-experimental designs to test the proposed pathways. Beyond these staged approaches, Miller et al. (2025) reviewed AI-driven advancements in evaluation, quality assessment, and teacher development, suggesting that examining AI-based tools for objective competence assessment and personalized feedback may complement traditional self-report measures.
