# Supplementary material for: Chinese primary school physical education teachers’ ongoing training and professional competence: an evidence mapping review and a hypothesis-generating conceptual framework
Source: Front Psychol. 2026 Aug 10;17:1901909. doi: 10.3389/fpsyg.2026.1901909 (PMC13502122; doi:10.3389/fpsyg.2026.1901909)
Supplement: Supplementary file 1 [file Data_Sheet_1.pdf]

Table S1. Summary of key prior reviews and theoretical frameworks relevant to ongoing training and professi

Number	Source	Scope/Focus
1	Musengamana & Hou (2025)	On-the-job training effectiveness
2	Ma (2025)	Teaching competence
3	Wu & Shen (2024)	Open class as professional learning
4	Zhang & Wong (2021)	School-based knowledge development
5	Dogan et al. (2015)	PLCs on science teachers
6	Opfer & Pedder (2011)	Teacher professional learning
7	Kunter et al. (2013)	Professional competence
8	Chen Y. (2015)	Teacher professional development in China
9	Gu & Wang (2006)	School-based research & professional learning

onal competence of primary school PE teachers

**Sample/Setting**	**Subject area**
Secondary school teachers (n = 3,976)	General (all subjects)
Primary school teachers (n = 179)	Chinese language
19 teachers (mixed levels)	PE (mixed levels)
31 secondary school teachers	General (all subjects)
14 reviewed articles	Science
Literature review	General (all subjects)
Theoretical model	General (all subjects)
Conceptual chapter	General (all subjects)
Theoretical model	General (all subjects)

**Key findings**
OJT significantly enhances teacher self-efficacy and human capital.
Identified five dimensions of teaching competence.
Open class bridges theory-practice gap.
Knowledge development characterized by self-knowledge and local knowledge.
PLCs enhance PCK and DCK; may shift practice toward inquiry-based approaches.
Effective professional learning must be sustained and intensive.
Competence comprises knowledge, beliefs, motivational and self-regulatory characteristics.
Inward school-based research cultivates autobiographical consciousness and teacher reflection.
Action Education model bridges theory-practice gap through lesson study cycles.

Gap identified
No primary or PE focus
Excluded PE specialists
Not specifically primary; not a review
Secondary focus only
Science focus; not PE or primary
Conceptual only; no empirical PE focus
Theoretical framework; not PE-specific
Theoretical; no empirical PE focus
Theoretical model; not PE-specific
