# Supplementary material for: Chinese primary school physical education teachers’ ongoing training and professional competence: an evidence mapping review and a hypothesis-generating conceptual framework
Source: Front Psychol. 2026 Aug 10;17:1901909. doi: 10.3389/fpsyg.2026.1901909 (PMC13502122; doi:10.3389/fpsyg.2026.1901909)
Supplement: Supplementary file 2 [file Data_Sheet_3.pdf]

Table S3 *Search terms used in WoS and Scopus for studies on ongoing training, primary school PE teachers in China, and professional competence.*

Step	Terms	Results
Database: Web of science		
1	TS=("on-going training" OR "on-going education" OR "continuing education" OR "ongoing training" OR "Learn*" OR "train*" OR "educat*")	4882098
2	TS= ("professional competence" OR "teaching competence" OR "teacher competence" OR "competence" OR "competency" OR "core competence" OR "core competency" OR "teacher competency development" OR "teacher professional development" OR "teacher competence" OR "professional development" OR "instructional leadership")	262091
3	TS= ("Physical Education Teacher" OR "teacher" OR "educator" OR "instructor" OR "school teacher" OR "lecturer" OR "coach" OR "mentor" OR "tutor" OR "teacher*" OR "school teacher*")	398363
4	TS= ("Chinese" OR "China" OR "the name of the provinces in China")	1614291
5	TS= ("primary school" OR "elementary school" OR "Junior school" OR "Kindergarten" OR "grade 1" OR "grade 2" OR "grade 3" OR "grade 4" OR "grade 5" OR "First Grade" OR "Second Grade" OR "Third Grade" OR "Fourth Grade" OR "Fifth Grade" OR "1st grade" OR "2nd grade" OR "3rd Grade" OR "4th Grade" OR "5th grade" OR "primary school" OR "School" OR "school*" OR "private" OR "public school" OR "grade school" OR "elementary education" OR "basic education")	1149068
6	1 AND 2 AND 3 AND 4 AND 5	1074
Database: Scopus		
1	TITLE-ABS-KEY ("on-going training" OR "on-going education" OR "continuing education" OR "ongoing training" OR "Learn*" OR "train*" OR "educat*")	8823971
2	TITLE-ABS-KEY("professional competence" OR "teaching competence" OR "teacher competence" OR "competence" OR "competency" OR "core competence" OR "core competency" OR "teacher competency development" OR "teacher professional development" OR "teacher competence" OR "professional development" OR "instructional leadership")	632146
3	TITLE-ABS-KEY ("Physical Education Teacher" OR "teacher" OR "educator" OR "instructor" OR "school teacher" OR "lecturer" OR "coach" OR "mentor" OR "tutor"	973693

	OR “teacher*” OR “school teacher*”)	
4	TITLE-ABS-KEY (“Chinese” OR “China” OR” the name of the provinces in China”)	2401370
5	TITLE-ABS-KEY (“primary school” OR “elementary school” OR “Junior school” OR “Kindergarten” OR “grade 1” OR “grade 2” OR “grade 3” OR “grade 4” OR “grade 5” OR “First Grade” OR “Second Grade” OR “Third Grade” OR “Fourth Grade” OR “Fifth Grade” OR “1st grade” OR “2nd grade” OR “3rd Grade” OR “4th Grade” OR “5th grade” OR “primary school” OR “School” OR “school*” OR “private” OR “public school” OR “grade school” OR “elementary education” OR “basic education”)	2465642
6	1 AND 2 AND 3 AND 4 AND 5	1142
