# Supplementary material for: Chinese primary school physical education teachers’ ongoing training and professional competence: an evidence mapping review and a hypothesis-generating conceptual framework
Source: Front Psychol. 2026 Aug 10;17:1901909. doi: 10.3389/fpsyg.2026.1901909 (PMC13502122; doi:10.3389/fpsyg.2026.1901909)
Supplement: Supplementary file 3 [file Data_Sheet_4.pdf]

Table S4. Extraction of descriptive information from the 29 included articles.

Number	Authors	Geographical location	Title	Journal
1	Xinyao Ma<br>-2025	China	Promoting primary school Chinese teachers teaching competence: A case study from China	International Journal of Teacher Education and Professional Development
2	Youjing Wang and Zhongtao Fan<br>-2025	China	The impact of communities of practice on professional development among Chinese teachers: A mixed-methods longitudinal study	Original Research
3	Shujuan Xu and Eng Tek Ong<br>-2025	China	Factors influencing professional development of double-qualified teachers in Guangxi Zhuang Autonomous Region, China	International Journal of Advanced and Applied Sciences
4	Jocelyn L. N. Wong and<br>Amy B. M. Tsui<br>-2014	China	How do teachers view the effects of school-based in-service learning activities? A case study in China	Journal of Education for Teaching
5	Irene Musengamana and Yuna Hou<br>-2025	China	Uplifting teachers’ human capital through on-the-job training: A determinant for teacher self-efficacy	Cogent Education
6	M. Kunter et al.<br>-2013	Germany	The Development of teachers’ professional competence	Springer
7	Chen Yuting<br>-2015	China	Teachers' professional development in China	Springer
8	Xiaolei Zhang and Jocelyn L.N. Wong （2021）	China	How do teachers perceive their knowledge development through engaging in school-based learning activities? A case study in China	Journal of Education for Teaching
9	Jinxiu Wu and Ping Shen<br>-2024	China	Facilitating teachers’ professional learning in open class: a qualitative case study in China	Humanities and Social Science Communications
10	Chunmei Yan and Chuanjun He	China	Enhancing part-time in-service teacher training programmes to facilitate rural	Springer

	-2011		teacher development in China	
11	Xiao Han and Zhuo Feng<br>-2013	China	School-based instructional research (SBIR): An approach to teacher professional development in China	Emerald Group Publishing Limited
12	Lingyuan Gu and Jie Wang<br>−2006	China	School-based research and professional learning: An innovative model to promote teacher professional development in China	Teaching Education
13	John Trent<br>−2020	China	Wither teacher professional development? The challenges of learning teaching and constructing identities across boundaries in China	Asia Pacific Journal of Education
14	Xiaojiong Ding, Lei Wang and Lingshuai Kong<br>-2023	China	The impact of local versus nonlocal professional development programs for teachers: Evidence from China's national teacher training project	Original Research
15	Yanping Fang<br>-2017	China	School-based teaching research<br>and lesson-case study in mediating the second-cycle curriculum reform in Shanghai	International Journal for Lesson and Learning Studies
16	Manhong K. Lai<br>-2009	China	Approaches to teacher development<br>in China: Hong Kong and Shanghai	International Journal of Educational Reform
17	Xue Han and Hequn Wang<br>-2010	China	Mathematics teachers' professional development opportunities in the curriculum reform of China	Research in Comparative and International Education
18	Chunmei Yan and Chuanjun He<br>-2015	China	'Short courses shouldn't be short-lived!' Enhancing longer-term impact of short English as a foreign language INSET initiatives in China	Professional Development in Education
19	Liang Huang et al.<br>-2019	China	Building school capacity for teacher learning in China	Professional Development in Education

20	Xiaojing Wang, Fan Fangb and Tariq Elyas<br><br>-2023	China	‘I have survived and become more confident': effects of in-service TKT-based training on primary school English teachers' professional beliefs and self-efficacy	Cambridge Journal of Education
21	Amy Ha et al.<br><br>-2004	China	Teachers' perceptions of in-service teacher training to support curriculum change in physical education: The Hong Kong experience	Sport, Education and Society
22	Selcuk Dogana Rose Pringleb and Jennifer Mes<br><br>-2015	USA	The impacts of professional learning communities on science teachers’ knowledge, practice and student learning: a review	Professional Development in Education
23	Zhongli Chen<br><br>-2025	China	Multimodal deep learning for sports teacher behavior analysis: design and evaluation of a<br><br>personalized continuing education<br><br>recommendation system	Scientific Reports
24	Ruoqi Li<br><br>-2023	Philippines	Research on the training of college physical education teachers from the perspective of career development	Frontiers in Sport Research
25	Fatkhur Rozi et al.<br><br>−2025	Indonesia	Sipemandu-GPT: A digital training model for 21st century PE teacher professionalism	Jurnal Eduscience
26	Tanja C. Sargent and Emily Hannum<br><br>-2009	China	Doing more with less: Teacher professional learning communities in resource-constrained primary schools in rural China	Journal of Teacher Education
27	V. Darleen Opfer and David Pedder<br><br>-2011	Britain	Conceptualizing teacher professional learning	Review of Educational Research
28	Jihong Yan and Xinyu Dai	China	Double marginalization: an ethnographic-ecological analysis of rural PE teachers’ professional	Frontiers

	-2025		development between urban and underdeveloped areas	
29	Luo et al. (2026)	China	Innovation of professional development model of college PE teachers driven by big data and the internet of things	International Journal of High Speed Electronics and Systems

Study design	Research participants	Data collection method	Data analysis techniques	Results/findings
Mixed methods research	179 primary school teachers	Investigation method, interview method	T-test, analysis of variance	The definition of competence The definition of teaching competence
Mixed-methods, longitudinal design	135 primary and secondary school teachers (physical education teachers)	Investigation method, interview method	Thematic analysis method	The practice community has significantly enhanced the professional development of primary and secondary school teachers in China.
Quantitative research	The sample population consisted of 250 double-qualified teachers, 100 educational administrators, and 50 policymakers.	Stratified random sampling	Structural equation model analysis	It was found that having physical and professional resources alongside continuous training and progressive educational policies enhances DQTs' professional development.
Mixed-methods, longitudinal design	The study consisted of 69 interviewees who teach in seven schools located at Guangzhou, Panyu, Shenzhen and Conghua, all of which are in urban areas of Guangdong Province in China.	Investigation method, interview method	Coding	The positive effects of school-based learning activities on professional growth of teachers.
Quantitative research	A total of 3,976 middle secondary teachers (1,035 men and 2,941 women)	Investigation method, interview method	Structural equation model analysis	Sustaining and enriching structured professional collaboration and development is not merely beneficial but essential for teacher quality and professional growth.
Not explicitly mentioned	Not explicitly mentioned	Not explicitly mentioned	Not explicitly mentioned	Teachers' professional competence is a diverse set of capacities, including knowledge, beliefs, and motivational and self-regulatory characteristics, that interact to determine how well teachers are able to meet the demands of their profession.
Not explicitly mentioned	Not explicitly mentioned	Not explicitly mentioned	Not explicitly mentioned	Schools should undertake "inward school-based research" situated in the lived context of their own schools. Such research aims to construct multiple platforms for dialogue to incorporate research in the exchange of ideas and the construction of cooperation among teachers so that they can begin to study their own and shared individual experiences. In such research, autobiographical consciousness can be cultivated. This way of school and inner-based research promises to contribute to teachers'
Qualitative study.	Thirty-one secondary school teachers	Using purposive sampling techniques, interview method	Coding	The findings showed that for teachers in China, school-based knowledge development was characterised by strong self-knowledge and local knowledge for enriching knowledge base of individual teachers
Qualitative research	Nineteen teachers	Observation method, interview method	Thematic analysis method	The findings underscore open class as a platform to bridge the gap between practice and theory.
Qualitative research	38 teachers	Reflective writing	Coding	The results suggest that part-time INSET programmes for rural teachers should be supplemented by various other forms of teacher development

				activities to meet the dual goals of developing such programmes and providing professional development for teachers.
Qualitative research	Chinese teacher	Narratives,<br>case Study,<br>class observation	Not explicitly mentioned	The practice of SBIR has played a critical role in helping teachers: (1) understand the concept of China's New Curriculum, (2) improve instructional strategies, (3) reflect on personal professional practice, and (4) achieve professional growth.
Not explicitly mentioned	Not explicitly mentioned	Not explicitly mentioned	Not explicitly mentioned	"Action Education", a school-based research and professional learning approach that has been widely implemented in schools in China for a number of years, is an innovative model that aimed at promoting teacher professional development on the basis of action learning.
Qualitative research	6 teachers	Interview method	Tools for discourse analysis developed by Fairclough	The teacher's identities were shaped by the learning they experienced during professional development.
Mixed-methods, longitudinal design	42685 teachers	Investigation method,<br>interview method	Multiple regression analysis	The study argues that local and nonlocal programs play different roles in teacher PD. While local programs are conducive to teachers' mastery of context-specific knowledge and skills that are directly connected to subject teaching, nonlocal programs are crucial for teachers' continuous professional development in the long term.
Qualitative research	Three secondary schools	Case narratives,<br>cross-case comparisons	Case narratives,<br>cross-case comparisons	The guideline for the Shanghai's second-cycle of curriculum reform has aimed from the start to build unity between curriculum development and teacher professional learning through the school-based teaching research (yanxun yitihua).
Qualitative research	Four secondary schools	Interview method,<br>purposive sampling	Not explicitly mentioned	The school needs to propose a systematic teacher development system.Teachers in both cities stressed the importance of reflection-on-action in the process of teacher development. Schools in the two cities emphasized collegiality as the major approach to teacher development.
Quantitative research	78 teachers	Investigation method	Descriptive statistics analyses, regression analyses	The results showed that the length of time the participants spent on attending school-based learning activities had a significant effect on their attitudes toward the effectiveness of the professional development and on their instructional practice.
Quantitative research	120 teachers	Investigation method	SPSS was utilized for the questionnaire analysis	A high level of motivation for professional development was evident; however, in contrast with the high motivation, the level of endorsement for the programme was rather low.
Qualitative research	Three primary schools,<br>two junior high schools,<br>one senior high school	Interview method	Coding	The study revealed that the effective strategies for promoting the case schools' capacity for teaching learning were, contingent on Chinese cultural and institutional contexts. The practical implications for building school capacity for teacher learning are discussed.

Qualitative research	42 English teachers	Focus groups and the teachers' reflective journals	Qualitative content analysis	The findings showed that the teachers experienced complex transformations in their professional beliefs and self-efficacy
Mixed-methods, longitudinal design	183 primary school teachers	Investigation method, interview method	Coding	The results showed that the participants felt that in-service training was needed to equip them to implement a physical education program in line with the curriculum reform.
Qualitative research	14 peer-reviewed articles	System search	Topic synthesis critical analysis	PLCs can help teachers increase their PCK and DCK; increases in PCK and DCK may facilitate the change in teacher practices from traditional into more inquiry-based approaches; science teachers collaboratively focusing on student learning in PLCs are more likely to change their practice; and studies do not embrace student learning as an essential feature of PLCs.
Quantitative research	124 physical education teachers	Multimodal data collection	Multimodal analysis	Multimodal behavior analysis can effectively identify specific development needs and generate targeted continuing education recommendations that significantly enhance sports teaching quality and professional development.
Qualitative research	Not explicitly mentioned	Not explicitly mentioned	Not explicitly mentioned	Universities must attach great importance to the cultivation of university physical education teachers and establish a comprehensive training system and incentive mechanism to ensure the sustainable development of the university physical education teacher team. At the same time, schools should strengthen the training of physical education teachers, extend the training time as much as possible, and shorten the training cycle as much as possible.
Mixed-methods, longitudinal design	The teachers who are members of KKGOMI in Jepara Regency	Investigation method, interview method, classroom Observation	Descriptive statistics average, expert scores, qualitative analysis	The Sipemandu-GPT-based training effectively enhances the competence and professionalism of physical education teachers.
Mixed-methods, longitudinal design	30 teachers	Investigation method, interview method	Coding, structural equation model, analysis	Findings indicate that professional learning communities penetrate to some of China's most resource-constrained schools but that their nature and development are shaped by institutional supports, principal leadership, and teachers'own initiative.
Qualitative research	Literature	Literature retrieval	Thematic analysis, conceptual analysis	Effective professional development will improve teacher instructional practices, which will result in improved student learning.Most research has concluded that activities that effectively support teachers' professional learning need to be sustained and inten sive rather than brief and sporadic.
Qualitative research	35 teachers	Interviews,	Thematic analysis	The findings demonstrate that rural HPE teachers' professional development exhibits distinctive ecological interactions: at the exosystem level, policy orientations and uneven resource allocation during urbanization constitute structural constraints; at the mesosystem level,

		field notes,<br>institutional records		school sports culture and colleague/family support networks serve as crucial mediating variables; while at the microsystem level, teachers' intrinsic commitment and professional autonomy play significant
Quantitative research	120 PE teachers	Internet of Things (IoT) ,wearables and edge, computing frame	Mixed-methods approach adapted from creswell	The professional development of university physical education teachers requires continuing education.

Relevancy to research
Medium
High
Medium
High
High
High
High
High
High
High

High
High
High
High
High
High
Medium
High
High

High
High
High
Medium
Medium
High
High
High
High

Medium
