# Supplementary material for: Chinese primary school physical education teachers’ ongoing training and professional competence: an evidence mapping review and a hypothesis-generating conceptual framework
Source: Front Psychol. 2026 Aug 10;17:1901909. doi: 10.3389/fpsyg.2026.1901909 (PMC13502122; doi:10.3389/fpsyg.2026.1901909)
Supplement: Supplementary file 5 [file Data_Sheet_5.docx]

**Supplementary Figure S1. PRISMA 2020 flow diagram of study selection**

**Identification of studies via other methods**

**Identification of studies via databases and registers**

Records identified from:

Google scholar (n = 8)

Records removed before screening:

Duplicate records removed (n =724)

Records removed for other reasons (n =6)

**Identification**

Records identified from*:

Web of science (n =1074)

Scopus (n =1442)

Total(n=2516)

Records excluded**

No English(n =43 )

Conference paper（n=173）

Book（n=25）

**Screening**

Records screened

(n =1786)

Reports not retrieved

(n =0)

Reports sought for retrieval

(n =8 )

Reports not retrieved

(n =0 )

Reports sought for retrieval

(n =1545)

Reports included:

relevant to our research questions(n =8)

Reports excluded:

Correction (n =2)

Abstract of the literature（n=2）

Literature Review（n=1）

Not relevant to our research questions（n=1519）

Reports assessed for eligibility(n =21)

Reports assessed for eligibility

(n =8)

**Included**

Studies included in review

(n =29 )
